# Gel Microparticles Based on Polymeric Sulfonates: Synthesis and Prospects for Biomedical Applications

**DOI:** 10.3390/ijms27010538

**Published:** 2026-01-05

**Authors:** Olga D. Iakobson, Elena M. Ivan’kova, Yuliya Nashchekina, Natalia N. Shevchenko

**Affiliations:** 1 Branch of Petersburg Nuclear Physics Institute Named by B.P. Konstantinov of National Research Centre “Kurchatov Institute”—Institute of Macromolecular Compounds, 199004 Saint-Petersburg, Russia; 2 Institute of Cytology, Russian Academy of Sciences, 194064 Saint-Petersburg, Russia

**Keywords:** drug carriers, microspheres, polysulfonates, reverse suspension polymerization

## Abstract

Polyelectrolyte microspheres based on a polymer containing sulfonate groups are considered promising drug delivery systems for encapsulating drugs and ensuring their prolonged release. In this study, gel microparticles based on various sulfonate-containing polymers were formed, and their potential as drug delivery systems was evaluated, particularly for the controlled administration of the cytotoxic anthracycline antibiotic doxorubicin and the antifungal drug fuchsine. An undeniable advantage of such gel microspheres is the presence in their structure of sulfonate groups localized both in the surface layer and in the volume. The main monomers used were styrene-4-sulfonic acid sodium salt and 3-sulfopropyl methacrylate potassium salt; spherical, porous microparticles were obtained via free-radical reverse suspension polymerization. Microsphere properties (size, porosity, pore structure, electrical surface properties, and swelling) were tailored by changing the nature of the sulfonate, using a comonomer (vinyl acetate or ethyl acrylate), adding a co-solvent, or modulating the crosslinker composition, which influenced drug loading efficiency (doxorubicin, fuchsine). The gel-like structure of the microspheres was confirmed, and the sulfonate groups were found to be distributed throughout both the surface layer and the internal volume of the microspheres. A comparison was also made with non-porous polymer particles containing sulfonate groups. The sorption capacity of the gel microspheres for doxorubicin was 2.2 mmol/g, significantly higher than the 0.4 mmol/g observed for the non-porous reference particles. The obtained values of doxorubicin sorption on gel microspheres are over 60 times higher than the values reported in the literature.

## 1. Introduction

The synthesis of polyelectrolyte-based polymer biomaterials is an intensively developing field over recent decades [1,2]. Particular interest is paid to polyelectrolytes derived from polymers containing sulfonate groups, as they find diverse biomedical applications as antibacterial coatings [2,3]; as mimetic interfaces mimicking sulfated glycosaminoglycans for stable capture of bone morphogenetic proteins [4]; as artificial synovial fluid for biomimetic aqueous lubrication and arthritis treatment [5]; and as drug delivery carriers for various diseases [6,7].

Sulfonated polyelectrolytes can be prepared from natural polymers. For instance, lignin-based lignosulfonate produced on a large scale [8,9] is used to encapsulate poorly water-soluble drugs and bioactive compounds such as thymol and its brominated derivatives, canthaxanthin, and others [10]. An undeniable advantage of polyelectrolytes based on natural polymers is their generally good biocompatibility. However, a notable drawback lies in the batch-to-batch variability of their physicochemical properties. In contrast, synthetic polyelectrolytes can be produced with highly reproducible physicochemical characteristics, although they typically exhibit inferior biocompatibility compared to their natural counterparts. Synthetic polyelectrolytes can be formulated as nano- and microparticles for drug delivery applications [11,12,13]. The synthesis of polyelectrolyte gel microparticles remains one of the most challenging and not fully explored areas [14].

Gel microparticles are interesting as promising substrates for the expansion and delivery of mesenchymal stem cells, as well as for embolization and treatment of various pathological conditions [15]. Gel microparticles present hydrophilic three-dimensional networks crosslinked via chemical or physical bonds. For biomedical use, such microparticles should exhibit good swelling in physiological solutions and biological fluids and be capable of adsorbing and sustaining the prolonged release of therapeutic agents. These properties came from their micrometer-scale dimensions and well-developed porous structure. Moreover, the presence of functional groups such as sulfonate, carboxyl, or hydroxyl enables tuning of both the swelling degree and sorption capacity of the gel microspheres. Their mechanical properties, particularly the Young’s modulus, should fall within the range of 0.1–100 MPa, since these values are close to the mechanical behavior of biological soft tissues [16].

To date, gel microparticles have been primarily synthesized using acrylate-based monomers bearing carboxylic acid groups [17] or from poly(vinyl alcohol) and its derivatives [18]. Synthesizing gel microparticles from monomers containing sulfonate groups is challenging, as the resulting polymers are typically water-soluble; to form microparticles based on them, it is necessary to use crosslinking agents and also to carry out polymerization in a limited volume. One effective approach for this purpose is inverse (reverse) suspension polymerization [19]. Inverse suspension polymerization is a heterogeneous process that begins with the formation of a water-in-oil emulsion. Organic solvents or oils, such as cyclohexane, heptane, or corn oil, serve as the continuous phase, while an aqueous phase containing dissolved hydrophilic monomers, crosslinkers, and an initiator acts as the dispersed phase. As the emulsion is thermodynamically unstable, it requires both constant mechanical agitation and surfactants with a hydrophilic–lipophilic balance (HLB) of 3 to 6. The resulting microparticles are characterized by the presence of sulfonate groups available for ion-ion interaction, distributed both on the surface and throughout the bulk of the particles. The chemical nature of these sulfonate groups (aliphatic or aromatic) significantly influences sorption capacity. We previously demonstrated that polyelectrolyte matrices based on monomers with sulfonate groups attached to aliphatic chains exhibit high porosity and swelling, yet show lower sorption capacity for metal ions compared to matrices containing sulfonate groups bonded to aromatic rings [20]. It is known that polymer spherical particles with a diameter greater than 1 µm do not have a toxic effect on the human body at particle concentrations less than 1 mg/mL [21]. Despite the range of studies, there remains insufficient systematic data on how to effectively tailor the physicochemical properties of sulfonated polyelectrolyte microspheres: their swelling ratio, functional group concentration, specific surface area, pore size, electrosurface properties, and sorption capacity toward various pharmaceutical compounds.

In this work, a series of cation-exchange microparticles was synthesized via inverse suspension polymerization using monomers bearing sulfonate groups of different chemical nature (aliphatic or aromatic). The influence of the crosslinker type and comonomer nature on the physicochemical properties of the resulting gel microparticles (swelling degree, sulfonate group concentration, zeta potential, and sorption capacity for doxorubicin and fuchsine) was systematically investigated. The structure of the synthesized nano- and microparticles was characterized by Fourier-transform infrared (FTIR) spectroscopy, optical microscopy, and scanning electron microscopy (SEM). Additionally, the cytotoxicity of the gel microparticles was evaluated.

## 2. Results

### 2.1. Synthesis of Gel Microspheres

**Scheme 1:**
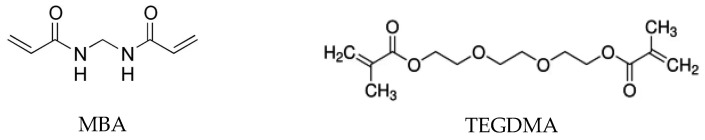
Structure of crosslinkers.

To study the influence of reaction mixture composition (comonomer nature, crosslinker type, and addition of a co-solvent) on the physicochemical properties of the resulting gel microspheres, reverse suspension polymerization was investigated. As primary functional comonomers containing sulfonate groups, the following were examined: sodium styrene-4-sulfonate (SSNa)-bearing an aromatic sulfonate group, and potassium 3-sulfopropyl methacrylate (SPM)-bearing an aliphatic sulfonate group. N,N′-methylenebis(acrylamide) (MBA) was used as the primary crosslinking agent (Scheme 1).

Initially, gel microspheres containing no additional comonomers were synthesized (PSS1 and PSPM1) (Table 1). To partially replace the ionogenic sulfonate groups with carbonyl groups, copolymerization of SSNa (or SPM) with vinyl acetate (VA) or ethyl acrylate (EA) was carried out (PSS2, PSS3, PSPM2, PSPM3) (weight ratio of SSNa(SPM):VA or SSNa(SPM):EA at 50:50). The simultaneous use of several crosslinkers in the synthesis of gel materials aligns with the principles of synergism and can affect the gel properties (e.g., swelling behavior, stability) [22,23], as well as enhance its functionality by incorporating different types of functional groups. Therefore, in this work, the synthesis of particles (PSS4, PSPM4) was also performed using a mixture of crosslinkers-N,N′-methylenebis(acrylamide) (MBA) and triethylene glycol dimethacrylate (TEGDMA) (50/50 wt.% ratio) (Scheme 1). It should be noted that introducing EA, VA, or the mixed crosslinker system into the reaction mixture reduced the yield of gel microspheres by 20%. The decrease in yield during copolymerization is due to the fact that in the process of reverse suspension polymerization, the aqueous phase contains the water-soluble monomer (SSNa or SPM), crosslinker, and water-soluble initiator (potassium persulfate) and is dispersed as droplets in a continuous nonpolar organic phase (cyclohexane here), stabilized by a mixed surfactant system (Tween 80 and Span^®^80). Due to its high hydrophilicity, SSNa (or SPM) remains almost entirely confined within the aqueous droplets, whereas the more hydrophobic comonomers (VA or EA) may partially enter into the organic phase. As a result, a fraction of VA/EA is lost from the aqueous droplets, leading to lower particle yields. Importantly, because polymerization is initiated exclusively by potassium persulfate within the aqueous droplets, copolymerization occurs only in these droplets that ensures the formation of copolymer microspheres. Homopolymerization of the VA/EA that migrates into the organic phase does not proceed, as no initiating radicals are present there. Thus, no homopolymer particles are formed, and the product consists of copolymer microspheres.

Conversely, the addition of the co-solvent undecyl alcohol was performed to enhance the stability of the formed emulsion; as a result, the yield of gel microspheres increased to 97% (PSS5 and PSPM5). Undecanol adsorbs at the water/oil interface, assisting the primary surfactant in reducing interfacial tension and thereby forming a more stable emulsion [24].

The compositions of the reaction mixtures and properties of the obtained particles discussed below (swelling degree, pore size, gel fraction etc.) are given in Table 1. The chemical formulas of the polymer chains that form microparticles are shown in Figure S1.

**Figure 1:**
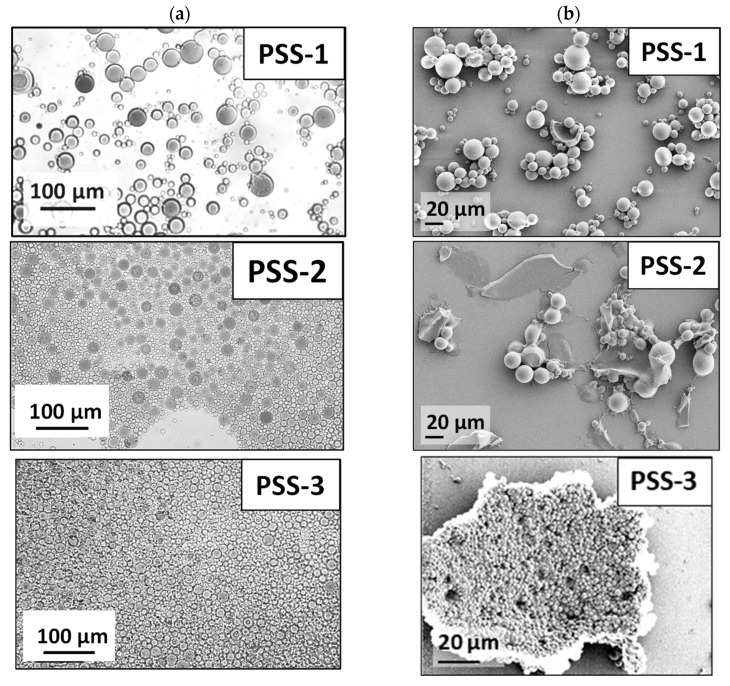

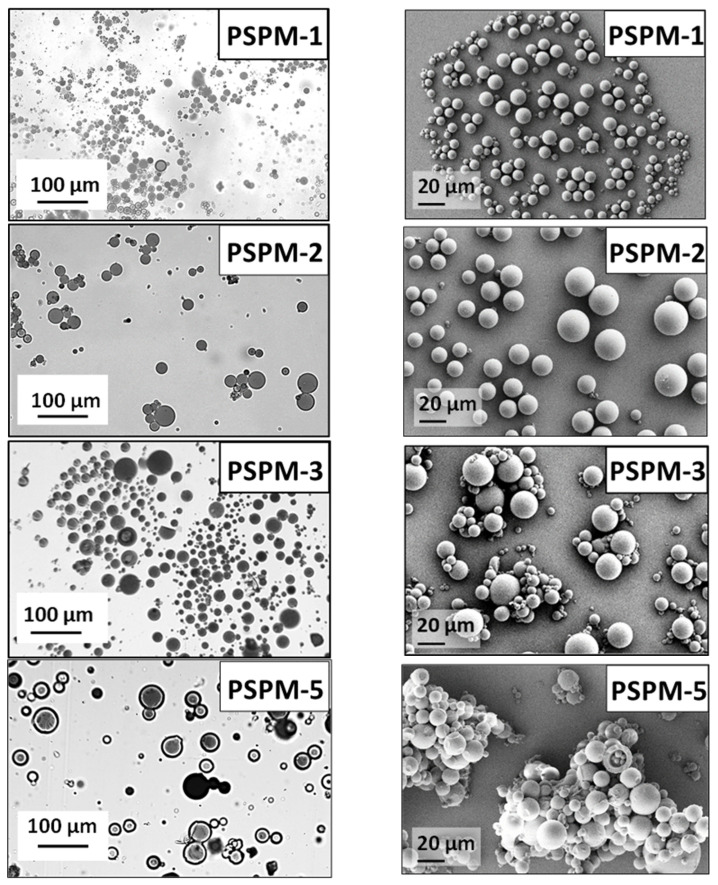
Images of microspheres based on P(SSNa) and P(SPM) obtained by optical microscopy (**a**) and scanning electron microscopy (**b**).

When comparing particles obtained using SSNa and SPM (samples PSS1 and PSPM1, Table 1), it is evident that smaller particles are formed in the case of PSPM1. This suggests that the emulsion in this case is formed of smaller and more stable droplets, consistent with SPM being more hydrophilic. It should be noted that, in all syntheses, a mixture of polyoxyethylene (20) sorbitan monooleate (Tween 80) and sorbitan monooleate (Span^®^ 80) was introduced into the reaction system as stabilizers, at a weight ratio of Tween 80:Span^®^ 80 = 16:84%. Tween 80 and Span^®^ 80 are nonionic surfactants that reduce interfacial tension at the water/cyclohexane boundary. The use of a mixture of these emulsifiers in the specified ratio gives an HLB value of 6.0, which corresponds to the upper limit of the HLB values recommended for obtaining stable “water in oil” emulsion system [25]. It is worth noting, that the stability of (W/O) emulsions using Span 80/Tween 80 in a weight ratio of 86/14 was more than 24 h (Figure S4). The incorporation of SPM into the aqueous dispersion medium provides additional electrostatic stabilization of the resulting water-in-oil emulsion droplets. In contrast, the SSNa monomer that also containing an aromatic sulfonate group does not exhibit comparable stabilizing effects. This difference is likely due to the higher conformational mobility of the aliphatic sulfonate group present in SPM monomer units, compared to the aromatic sulfonate group in SSNa monomer units [26]. Changing the composition of the reaction mixture (introducing comonomers, using a mixture of crosslinkers, or adding a co-solvent) leads to a decrease in the size of the formed particles for the PSS series, whereas for the PSPM series, it results in an increase in particle size (Table 1, Figure 1 and Figure S2). This trend can be explained as follows: reducing the concentration of SSNa in the aqueous phase and increasing the concentration of VA (or EA) enhances the effective structural stabilization of the water-in-oil emulsion, since VA (or EA) molecules are smaller and likely orient more efficiently at the water/oil interface. Conversely, reducing the concentration of the more hydrophilic SPM molecules (relative to VA or EA) in the aqueous phase diminishes the electrostatic stabilization of the water-in-oil emulsion.

In all cases, crosslinked porous particles were obtained (the gel fraction exceeded 85% for all samples, Table 1). Notably, the introduction of comonomers, as well as the use of the co-solvent (undecyl alcohol), leads to a reduction in specific surface area. In contrast, employing a mixture of crosslinkers positively affects the specific surface area, increasing it by 25–35% (Table 1). This result is clearly attributable to the formation of a crosslinked gel microsphere structure in the presence of the mixed crosslinker system, where polymer chains exhibit greater mobility due to the longer spacer in the TEGDMA structure. Using MBA alone results in a denser crosslinking network.

Optical microscopy images (water-swollen particles contrasted with methylene blue) and SEM images (freeze-dried particles) Figure 1 and Figure S2) confirm that spherical microparticles were obtained in all cases, with sizes up to 20 μm in the dried state.

**Figure 2:**
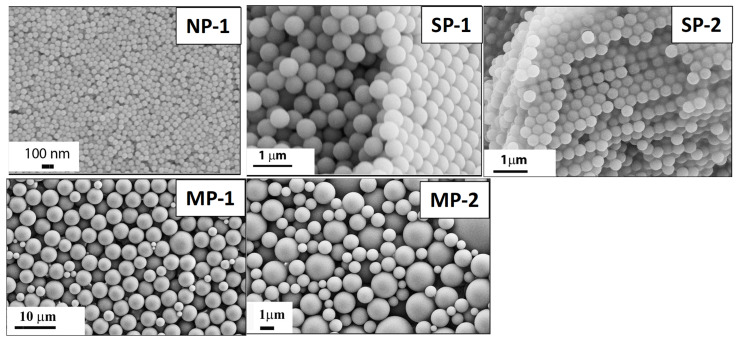
Images of nano- and microparticles of comparison obtained by scanning electron microscopy.

As reference samples, nano- and microparticles based on styrene were synthesized, with either SSNa or SPM introduced into the reaction system as the functional comonomer. These model nano- and microparticles have sulfonate groups localized exclusively in the surface layer, as their cores consist of hydrophobic polystyrene chains. Nanoparticles were synthesized via emulsifier-free emulsion copolymerization of styrene with SSNa. As a result, nanoparticles NP1 with a diameter of 70 nm were obtained (Table 2, SEM, Figure 2). Additionally, submicron particles SP1 and SP2 with diameters of 520 nm and 310 nm, respectively, were also prepared using the emulsifier-free emulsion copolymerization method. Microparticles MP1 and MP2 were synthesized by dispersion polymerization, with SSNa- or SPM-derived units, respectively, localized in their surface layers.

### 2.2. FTIR Spectra

**Figure 3:**
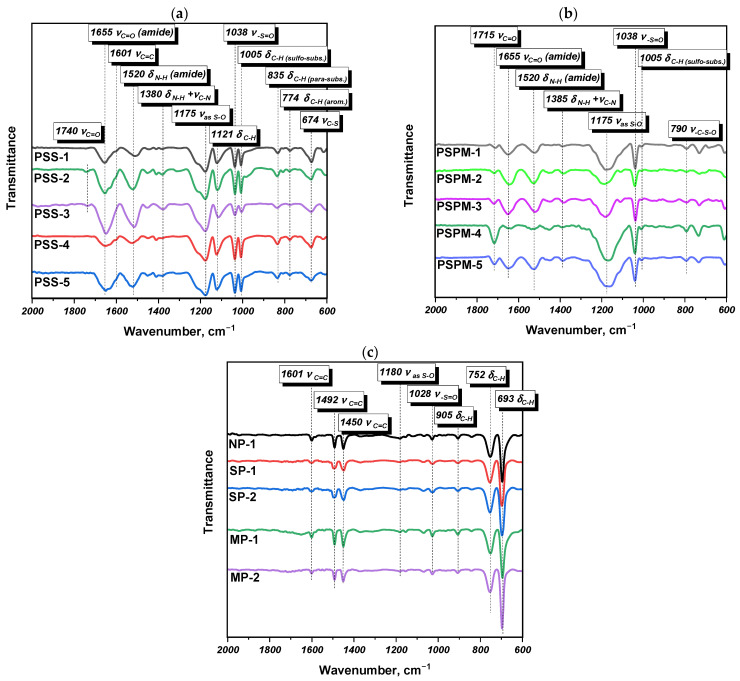
FTIR-spectra of powders of microspheres based on P(SSNa) (**a**), P(SPM) (**b**) and nano- and microparticles of comparison (**c**).

Analysis of the FTIR spectra recorded for the microsphere powders confirms the inclusion of the monomers in the polymer chain (Figure 3).

Particles synthesized on the basis of SPM are characterized by stretching vibrations of the C=O bond at 1715 cm^−1^ [27] and stretching vibrations of -C-S-O at 790 cm^−1^ [28]. Styrene sulfonate in the chemical structure is evidenced by the bands of C=C stretching vibration of aromatic rings at 1601 cm^−1^, in-plane vibrations of 1,4-disubstituted benzene ring at 1121 cm^−1^, in-plane deformation vibrations of C-H in the sulfo-substituted aromatic ring at 1005 cm^−1^, CH out-of-plane vibrations for para-disubstituted benzene at 835 cm^−1^ [29], the band at 774 cm^−1^ corresponds to the deformation vibrations of δ(C-H) of the aromatic ring [20] and 674 cm^−1^ to stretching vibrations of C-S [29].

The presence of sulfo groups in the particles is indicated by asymmetric stretching vibrations of –SO_3_ at 1175 cm^−1^, stretching vibrations of –S=O in –SO_3_¯ at 1038 cm^−1^ [29]. The inclusion of the MBA crosslinker in the structure of the crosslinked particles can be confirmed by the presence of bands of C=O stretching vibrations of amides (MBA) (amide I) at 1655 cm^−1^, N-H deformation vibrations (MBA) (amide II) at 1520 cm^−1^, and conjugated NH deformation vibrations and CN stretching vibrations at 1380–1385 cm^−1^ (amide III) [27].

EA and VA have functional groups associated with vibrations of the C=O bond, therefore the identification of these comonomers in the copolymer with SPM is not possible, however, in the structure of the copolymer with SSNa, the inclusion of VA and EA produces a band of about 1740 cm^−1^ (ν_C=O_).

The FTIR spectra of reference particles contain key FTIR bands for polystyrene: at 1601 cm^−1^, 1492 cm^−1^, and 1450 cm^−1^ due to aromatic C=C stretching; and at 905 cm^−1^, 752 cm^−1^ and 693 cm^−1^, related to aromatic C-H out-of-plane bending. Intense bands characteristic of polystyrene overlap many characteristic bands of sulfo groups due to their low content in NP1, SP-1, SP-2, MP-1, and MP-2 particles. However, sulfo groups in the abovementioned particles are evidenced by asymmetric stretching vibrations of –SO_3_ at 1180 cm^−1^ [29].

### 2.3. Study of Electrical Surface Properties

**Figure 4:**
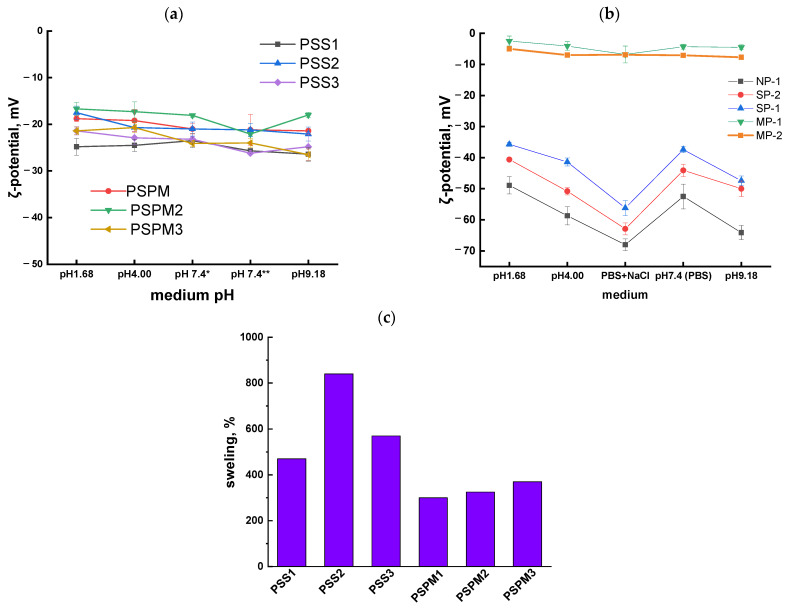
Electrosurface properties of gel microparticles (**a**) and comparison particles (**b**), as well as swelling of gel microparticles (**c**). * PBS + NaCl (0.9 M) medium; ** PBS medium.

The electrosurface properties of the gel microspheres were investigated depending on dispersion medium composition and pH: in PBS (phosphate-buffered saline), in PBS + NaCl (0.9 M), and across a pH range from 1.68 to 9.18. It was found that the zeta potential of all gel microspheres remained within the range of −15 to −25 mV, regardless of the dispersion medium composition (Figure 4a). The gel microspheres exhibited aggregation stability under all tested conditions (adding salt, increasing the concentration of H^+^ or OH^−^ ions), indicating that the polymer chains bearing ionogenic functional groups are conformationally flexible. Consequently, when a portion of these ionogenic groups forms ion pairs with oppositely charged counterions, functional groups localized within the gel volume reorient and migrate toward the slipping plane, thereby maintaining a relatively constant zeta potential. This conclusion is supported by swelling data: the degree of swelling for the gel microspheres ranged from 300% to 840% (Figure 4c). Clearly, the conditions of the reverse suspension copolymerization (i.e., variations in reaction mixture composition) significantly influence the swelling behavior of the microspheres, however, the surface concentration of sulfonate groups appears to remain effectively constant, leading to only a weak dependence of zeta potential on the dispersion medium composition.

The concentration of sulfonate groups in the gel microspheres was determined by two independent methods (conductometric titration and ion-exchange capacity) and both yielded consistent results. This agreement indirectly confirms the high permeability of the gel microspheres and the rapid ion-exchange kinetics occurring within their network (Table 1).

In contrast, the zeta potential of the comparison particles showed an opposite trend depending on the dispersion medium (Figure 4b). All reference particles contain sulfonate groups in their surface layer only, and their surface concentration is approximately three orders of magnitude lower than that of the gel microspheres. Moreover, these particles are non-swelling in aqueous media. The lowest zeta potential values (−5 mV and −7 mV) were observed for microparticles MP1 and MP2, respectively The presence of the steric stabilizer polyvinylpyrrolidone in the surface layer of these particles in addition to the sulfonate groups, not only explains weak negative zeta potential of samples MP1 and MP2, but also independence of the zeta potential on both dispersion medium composition and pH. The zeta potential of nano- and submicron comparison particles is significantly greater than that of gel microparticles, as well as significantly depends on the composition of the dispersion medium (Figure 4b).

Notably, because the surface of these comparison particles contains a limited number of sulfonate groups, increasing the ionic strength of the medium (to a physiologically relevant level, comparable to blood plasma) leads to extensive formation of ion pairs. Under these conditions, nearly all ionogenic surface groups become involved in ion pairing and are effectively localized at the slipping plane, resulting in a pronounced decrease in zeta potential as low as −70 mV for nanoparticle sample NP1.

Thus, the investigation of electrosurface properties demonstrated that the gel microspheres exhibit nearly constant zeta potential values, regardless of the composition of the dispersion medium. Even under conditions mimicking physiological fluids, the gel microspheres remain aggregation stable due to their high concentration of sulfonate groups (≈2 mmol/g) and their swellable gel network structure.

### 2.4. Study of Sorption Capacity

**Scheme 2:**
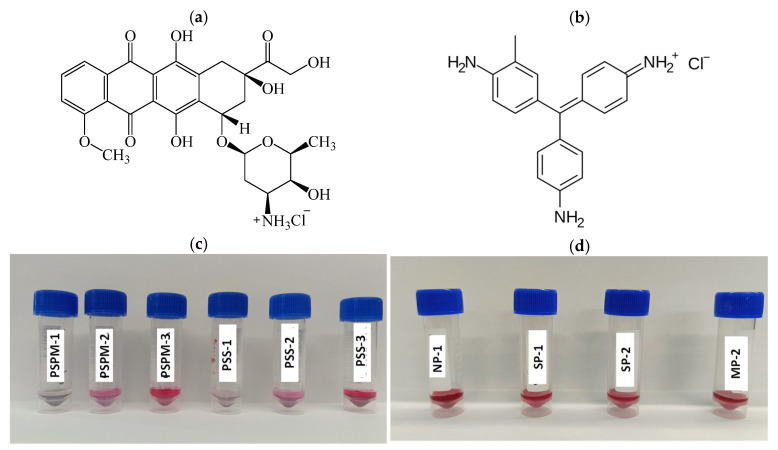
Structural formulas of doxorubicin (**a**) and fuchsine (**b**), photograph of the supernatant liquid during sorptiocn of fuchsine onto gel microspheres (**c**) and comparison nano- and microparticles (**d**).

It is demonstrated the influence of the physicochemical properties of gel microparticles on their sorption capacity toward the pharmaceutical compounds doxorubicin and fuchsine (Scheme 2).

**Figure 5:**
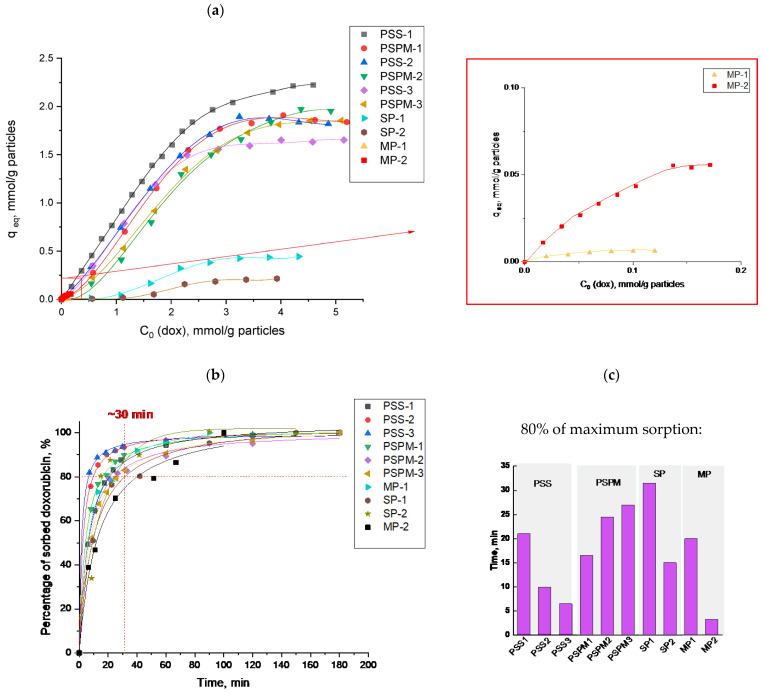
Sorption capacity of gel microspheres and comparison particles in relation to doxorubicin (**a**), sorption kinetics (**b**,**c**).

The highest sorption capacity for doxorubicin was observed for PSS1 gel microspheres (2.2 mmol/g), which contain no VA or EA units in their polymer network. Incorporating VA or EA comonomer units into the microsphere structure reduced the sorption capacity by approximately 25% (Figure 5). Notably, the sorption capacity of SPM-based gel microspheres was found to be independent of the chemical composition of the polymer chains and remained consistently around 1.85 mmol/g. Moreover, no direct correlation could be established between the sorption capacity of the gel microspheres and either their degree of swelling or the concentration of sulfonate groups (Figure 4c, Table 1). For instance, PSS2 microspheres exhibit the highest swelling ratio (among PSS1, PSS2, and PSS3) and the highest surface concentration of functional groups (Table 1, mmol/m^2^). One might therefore expect them to display the highest sorption capacity. However, experimental results showed that PSS2 microspheres sorbed no more than 1.86 mmol/g of doxorubicin. It was observed that sorption capacity correlates directly with pore volume. PSS1 microspheres possess the largest pore volume and simultaneously demonstrate the highest sorption capacity for doxorubicin. Thus, efficient sorption of doxorubicin requires a well-developed porous structure within the gel microspheres, combined with an optimal degree of swelling (not exceeding 450%).

As expected, the absence of sulfonate groups within the bulk of the comparison particles led to a significant reduction in drug sorption capacity (Figure 5). For submicron reference particles SP1 and SP2, the sorption capacity dropped by a factor of five compared to the gel microspheres (Table 3, Figure 5a). Furthermore, reducing the sulfonate group concentration in SP2 particles by 2.5 times (relative to SP1) resulted in a proportional 2.5-fold decrease in doxorubicin sorption capacity.

The lowest sorption capacities were observed for the micron-sized reference particles MP1 and MP2-approximately 1000-fold and 100-fold lower, respectively, compared to the gel microspheres. Such reduced values are clearly attributed to the fact that, in addition to sulfonate groups, the surface layer of these particles also contains polyvinylpyrrolidone (PVP) polymer chains. As a result, the accessibility of sulfonate groups for interaction with doxorubicin molecules is significantly hindered. It should also be noted that aliphatic sulfonate groups owing to their greater conformational mobility interact more effectively with doxorubicin molecules than their aromatic counterparts.

The kinetics of doxorubicin sorption were Investigated for both gel microspheres and reference particles. Although the maximum sorption capacities of the gel microspheres fall within a relatively narrow range (1.65–2.22 mmol/g), their sorption kinetics differ significantly. Incorporating VA or EA comonomer units into the PSS-based gel microspheres markedly accelerates the sorption process (Figure 5c). This is likely because SSNa-derived units exhibit limited conformational flexibility; the introduction of more flexible VA or EA units creates a more favorable environment for faster coordination of doxorubicin molecules with the polymer chains. In contrast, adding VA or EA comonomers to PSPM-based gel microspheres slows down sorption kinetics, as it reduces the effective ion–ion interactions between the mobile aliphatic sulfonate groups and doxorubicin molecules. For the reference particles, sorption kinetics correlate directly with the surface concentration of sulfonate groups in SP1 and SP2. Aromatic sulfonate groups in the surface layer leads to slower sorption, likely due to the restricted conformational mobility of these rigid aromatic groups. Interestingly, although MP2 particles sorb 10 times more doxorubicin than MP1 particles, the sorption process for MP2 is the fastest among all samples and reaches equilibrium in less than 4 min. This indicates that the fastest ion–ion interaction occurs between aliphatic sulfonate groups and doxorubicin molecules.

The experimental sorption data were analyzed using the Langmuir theoretical model, which assumes adsorption onto a homogeneous surface with formation of monolayer. The applicability of this model was assessed by evaluating adjusted coefficient of determination (adj. R^2^) as a convergence criterion between experimental and theoretical data (Table 3). As shown in the table, the Langmuir model is only reasonably applicable to the reference particles (e.g., SP1, SP2) for describing the sorption mechanism.

Clearly, the Langmuir model is not suitable for describing sorption processes that occur not only at the surface but also throughout the bulk of the material. Among the models tested, the Freundlich isotherm provided the best fit for the gel microspheres (R^2^ = 0.88–0.96 for samples PSS1, PSS2, PSS3, PSPM1, and PSPM3), suggesting that drug adsorption occurs on a non-uniform surface with active sites exhibiting varying adsorption energies.

In addition, the sorption capacity toward fuchsine was also investigated (Table 4). Structurally, fuchsine contains two additional amino groups compared to doxorubicin (Scheme 2). Consequently, the sorption capacity of PSS-based gel microspheres for fuchsine is approximately 1.6 times higher than for doxorubicin. However, despite PSS2 and PSS4 microspheres exhibiting the highest degrees of swelling, their fuchsine sorption capacity does not exceed 3.3 mmol/g. Notably, PSS4 microspheres also possess the largest mesopore volume. It appears that, for PSS4, the extremely high swelling ratio (845%) leads to excessive pore volume, which in turn promotes desorption of drug molecules. Thus, two critical conditions must be simultaneously satisfied for efficient drug sorption. From the one hand, the degree of swelling must not be excessive, for example, for PSS-based microspheres it should not exceed 450%. On the other hand, the gel network must contain a sufficient volume of mesopores-at least 0.004 m^3^/g. A similar trend was observed for P(SPM)-based gel microspheres in fuchsine sorption. The highest sorption capacity was also recorded for PSPM1, which contains no additional functional comonomers.

Kinetic studies of fuchsine sorption revealed that only PSS1 and PSPM1 microspheres achieve complete adsorption of fuchsine into their bulk within 24 h (Scheme 2c). In contrast, when VA or EA units are incorporated into the polymer chains, either not all fuchsine molecules penetrate the microsphere interior or concurrent desorption takes place, preventing full retention of the dye.

A study of the sorption of fuchsine on comparison particles showed that sorption on them was ineffective: the supernatant liquid remained intensely colored after 24 h (Scheme 2d).

### 2.5. Cytotoxicity Study

**Figure 6:**
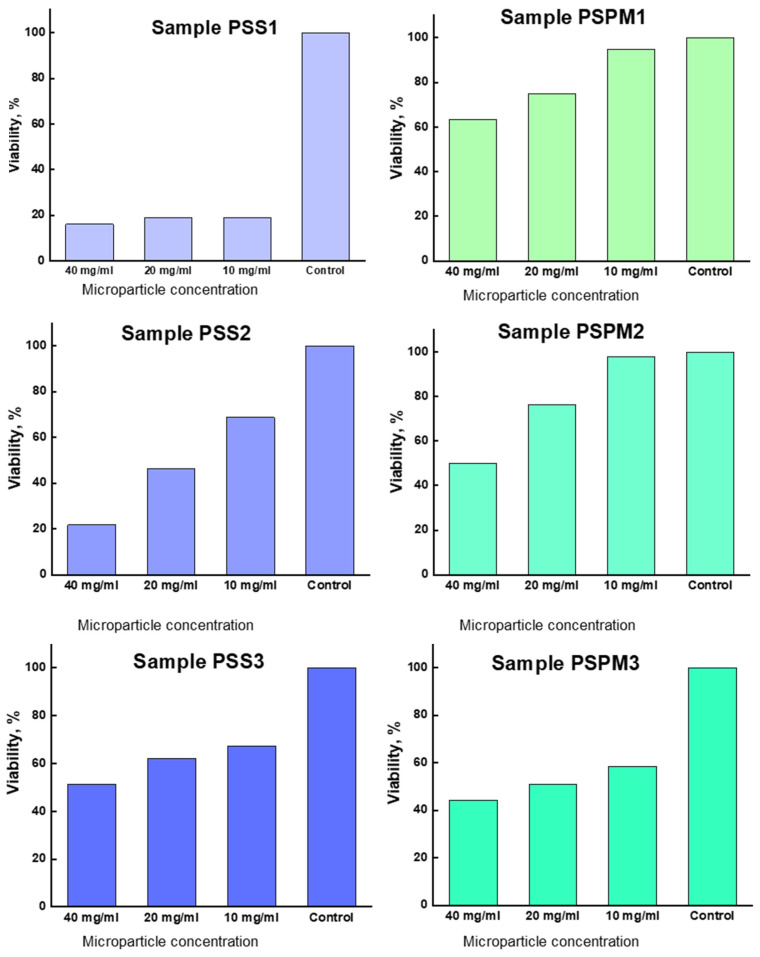
Results of the MTT test for FetMSCs cells in a nutrient medium during 3 days of cultivation after incubation with experimental gel microparticles.

The cytotoxicity of the resulting gel microspheres was tested using the MTT assay. As shown in Figure 6, the P(SPM)-based gel microspheres exhibited the lowest cytotoxicity compared to P(SSNa)-based particles. The introduction of VA units into the P(SPM) polymer chain increased the percentage of viable cells to 98%, at a microsphere concentration of 10 mg/mL. The introduction of EA units into the P(SPM) polymer chain in turn reduced µthe percentage of viable cells to 50%, with survival being only slightly dependent on the microsphere concentration (in the range of 10 to 40 mg/mL). The PSS1 gel microspheres were found to reduce the percentage of viable cells to 20%, even at a concentration of 10 mg/mL. The introduction of functional comonomers VA or EA into PSS2 and PSS3 gel microspheres increased cell viability to 68%, even at a microsphere concentration of 10 mg/mL. The MTT assay data (Figure 6) were consistent with optical microscopy data (See SI, Figure S3). It is obvious that introduction of a comonomer into the chain of polymeric sulfonates reduces the toxicity of microparticles regardless of the nature of the sulfonate group in the polymer (aliphatic, aromatic group).

The cytotoxicity results alone do not justify concluding that gel microspheres are unsuitable for drug delivery. The observed reduction in cell viability in the presence of P(SSNa)-based gel microspheres may be attributed to the presence of small particles (<1 µm), as it was shown that the cytotoxicity caused by nano-level polystyrene-based particles (<1 μm) is more serious than that of micro-level ones [21,30]. It should be noted that a univocal answer about the reasons for changes in cell viability can only be given as a result of additional size-dependent future studies for a specific case.

Therefore, further investigation is essential to fully elucidate the interactions between cells and polymer gel microspheres and to assess their true potential for drug delivery applications.

## 3. Discussion

**Scheme 3:**
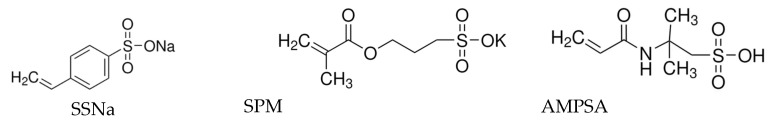
Structure of monomers.

The main aim of this study was the synthesis of gel microspheres based on sulfonated monomers, followed by an investigation of their sorption properties toward pharmaceutical compounds and an assessment of their cytotoxicity. Currently, the scientific literature lacks sufficient data describing the reverse suspension polymerization of sulfonated monomers. The most commonly used types of monomers containing sulfonate groups are summarized in Scheme 3.

A key challenge in this field comes from the absence of a unified theoretical framework explaining how emulsifiers of different nature influence the stabilization of water-in-oil droplets during polymerization. Moreover, to date, there is no clear understanding of whether it is feasible to produce microspheres with a unimodal particle size distribution.

Branger and coauthors demonstrated the possibility of synthesizing cross-linked microspheres based on vinylbenzyliminodiacetic acid via suspension polymerization [31] Choi described the synthesis of sodium polyacrylate-based polymeric microparticles of 30–100 µm in size using inverse suspension polymerization [32].

Among the few studies devoted to synthesizing sulfonate-containing gel microspheres via inverse suspension polymerization is the study by Khazdooz et al. [33]. This study reports a method for synthesizing gel microspheres from styrene sulfonate and 2-acrylamido-2-methyl-1-propanesulfonic acid (AMPSA). The authors demonstrated that microspheres based on AMPSA exhibit the highest adsorption capacity toward lactoferrin protein with a maximum adsorption capacity of 204 mg/g. Additionally, the AMPSA monomer was incorporated into polyvinyl chains using suspension polymerization [12]. The resulting cross-linked microspheres displayed methylene blue (used as a doxorubicin model compound) adsorption capacities of 20.7 mg/g.

In our work, we investigated how the composition of the reaction mixture influences the diameter and physicochemical properties of the resulting gel microspheres. Emulsion polymerization methods (including emulsion, emulsifier-free emulsion polymerization) for synthesizing sulfonate-functionalized polymer particles sized 100 nm to 3 µm are extensively reported in the literature [34,35]. Both methods yield particles in which sulfonate groups are localized only at the surface layer. For comparison purposes, we also employed emulsion polymerization and emulsifier-free emulsion polymerization. Our results show that the diameter of gel microspheres can be varied within a range of 1–20 µm. The narrowest size distribution is achieved when copolymerizing sodium styrene sulfonate (SSNa) with ethyl acrylate (EA), or when incorporating undecanol as a co-solvent. It was found that using SSNa as the sulfonate-containing comonomer allows formation of gel microparticles with swelling degrees ranging from 345% to 845% and mesopore volumes from 0.001 to 0.008 m^3^/g.

The primary question addressed in this study concerns how the nature of the sulfonate group affects the physicochemical properties of gel microspheres. To this aim, we synthesized two series of gel microspheres: one based on SSNa (bearing aromatic sulfonate groups, see Scheme 3), and another based on SPM (containing aliphatic sulfonate groups). Results show that the physicochemical properties of SSNa-based microspheres (e.g., sulfonate group concentration, swelling degree, mesopore volume, and drug adsorption capacity) can be tuned across a broader range of values. Presumably, this results from differences in conformational flexibility between aliphatic and aromatic sulfonate groups [26]. The lower conformational mobility of sulfonate moieties of SSNa influences not only the swelling behavior of the gel microspheres but also ion–ion interactions with cationic drugs. Consequently, such microspheres achieve a doxorubicin adsorption capacity of 2.2 mmol/g (1273 mg/g) that is over 60 times higher than the values reported in [12]. It should be noted that incorporating VA or EA units into the polymer chains reduces the sorption capacity for both doxorubicin and fuchsine by approximately 25%. The experimental data indicate that the primary driver of sorption is ion–ion interactions between sulfonate groups localized in structure of the gel microspheres and protonated amino groups of the drug molecules. Consequently, the introduction of non-ionic comonomers that reduced the amount of sulfonate groups diminishes sorption capacity. This study demonstrates that the sorption capacity toward cationic drugs correlates strongly with two key physicochemical properties of the gel microspheres: pore volume and degree of swelling. To maximize sorption, microspheres should exhibit a pore volume of at least 0.004 m^3^/g, while maintaining a moderate swelling degree not more than 450%. For instance, PSS-4 gel microspheres possess a favorable pore volume (0.008 m^3^/g) but exhibit excessive swelling (845%), resulting in the lowest fuchsine sorption among the tested samples. Notably, these microspheres also display rapid drug desorption. Thus, effective sorption of cationic drugs requires structural features that impose steric hindrance to rapid desorption, both at the surface and within the gel network.

It is worth noting that our study identified optimal synthesis conditions for SSNa- or SPM-based gel microspheres aimed at adsorbing cationic drugs like doxorubicin or fuchsine, with the highest adsorption capacities observed for microspheres bearing aromatic sulfonate groups. Nevertheless, the obtained microspheres may prove less effective for other drugs or proteins, warranting further investigation. Khazdooz’s results indicate higher lactoferrin adsorption capacities for zwitterionic sulfonate monomer-based microspheres (e.g., AMPSA, containing aliphatic amino groups) [33].

Comparing the physicochemical properties of the synthesized gel microspheres with those of reference particles revealed clear advantages for gel microspheres. Firstly, sulfonate group concentrations in gel microspheres can be up to three orders of magnitude higher than in reference particles. Such high concentration ensures both efficient drug adsorption and aggregation stability in dispersion media resembling physiological solutions. Secondly, the gel structure of microsphere imparts favorable mechanical properties: limited swelling renders the microspheres “soft” relative to biological tissues. Our experimental findings are consistent with literature data [36]. For instance, Elmesallamy reported methylene blue adsorption capacities not more than 18 mg/g for particles with surface-localized sulfonate groups. Thus, gel microspheres offer two decisive advantages over reference particles: a three-order-of-magnitude increase in sulfonate group concentration, enabling high drug adsorption capacities; and a “soft” gel structure that preserves microsphere integrity and functionality across various physiological fluids.

Cytotoxicity assessments revealed that PSPM and P(SPM-VA)-copolymer-based gel microspheres hold the greatest promise for drug delivery applications, exhibiting nearly 100% viability with the FetMSCs (mesenchymal stromal cell) line.

It should be noted that the primary limitation of this study is the relatively broad particle size distribution of the resulting gel microspheres, particularly when compared to those obtained via dispersion polymerization. Nevertheless, inverse suspension polymerization enables the synthesis of gel microspheres from hydrophilic monomers, conferring unique physicochemical properties, including high swelling in physiological media, biocompatibility, and high sorption capacity for various drugs. Further investigation into the fundamental mechanisms of inverse suspension polymerization is expected to support the development of a predictive theoretical framework, that allows the synthesis of gel microspheres with a polydispersity index below 0.1.

## 4. Materials and Methods

### 4.1. Chemicals

Monomers: Styrene-4-sulfonic acid sodium salt (SSNa), 3-sulfopropyl methacrylate potassium salt (SPM), vinyl acetate (VA), ethyl acrylate (EA), styrene (St) and methacrylic acid (MAC) were purchased from Merck KGaA (Darmstadt, Germany); St, MAA, VA and EA were vacuum distilled before use according to standard procedures. Crosslinkers: N,N′-methylenebis(acrylamide) (MBA) and triethylene glycol dimethacrylate (TEGDMA) were purchased from Merck KGaA (Darmstadt, Germany) and used as received. Initiators: potassium persulfate (K_2_S_2_O_8_) produced by Vekton LLC (Saint Petersburg, Russia) was purified by recrystallization from water, azobisisobutyronitrile (AIBN) produced by Vekton LLC (Saint Petersburg, Russia) was purified by recrystallization from ethanol, and 4,4′-azo-bis(4-cyanisovaleric acid) (CVA) (Merck KGaA, Darmstadt, Germany) was used as received. Emulsifiers: polyoxyethylene (20) sorbitan monooleate (Tween 80, Ferak Berlin GmbH, Berlin, Germany), sorbitan monooleate (Span^®^ 80, Merck KGaA, Darmstadt, Germany), and polyvinylpyrrolidone (PVP) with a molecular weight (MM) of 35,000 from Sigma-Aldrich (Darmstadt, Germany) were used without additional purification. Solvents: cyclohexane (Vekton LLC, Saint Petersburg, Russia) was used without further purification; deionized water with a conductivity of not more than 0.12 μS cm^−1^ (“AQUARIUS-M” deionization system, Moscow, Russia) was used to prepare all solutions and carry out polymerization. Property characterization reagents: NaCl, HCl, and NaOH (Vekton LLC, Saint Petersburg, Russia) and doxorubicin and fuchsine (from Merck KGaA, Darmstadt, Germany) were used as received.

### 4.2. Synthesis of Polyelectrolyte Microspheres via Inverse Suspension Polymerization

The polymerization was carried out in an argon atmosphere. The synthesis was carried out in a 100 mL flask equipped with a reflux condenser and mechanical stirrer. A weighed sample of monomers (SSNa, SPM, SSNa(SPM):VA or SSNa(SPM):EA at 50:50 wt. %, 0.5 g), crosslinkers (MBA or MBA:TEGDMA = 50:50 wt. %, 0.25 g), initiator (K_2_S_2_O_8_, 4.5 wt.% to monomers), and the calculated amount of deionized water were first loaded into a flask and stirred until dissolved at a temperature not exceeding 40 °C for 20 min. In parallel, an emulsion of emulsifiers (Tween 80: Span^®^ 80 = 16:84 wt.%) in cyclohexane was prepared using an ultrasonic bath (Sapphire, Moscow, Russia). In experiments with a co-solvent, 10% vol. of cyclohexane was replaced by undecyl alcohol. The ratio of water to oil phase was 47:53 wt.%. The two phases were combined in a flask and stirred for 20 min at 50 °C. The reaction temperature was then increased to 70 °C. Completion of the polymerization reaction was indicated by a characteristic increase in the viscosity of the system. The reaction system was then cooled, and the particle dispersion was purified from unreacted reagents and reaction byproducts by repeated washing with an aqueous ethanol solution (40% vol.), followed by freeze-drying (FreeZone Freeze Dry System, Labconco Corporation, Kansas City, MO, USA) of the washed particles. For the sample abbreviation, please see the Table 1.

### 4.3. The Synthesis of Comparison Particles

The synthesis of NP-1 comparison particles was carried out by emulsifier-free emulsion copolymerization. The synthesis was carried out in a 100 mL flask equipped with a reflux condenser and a mechanical stirrer. 50 mL of H_2_O and 0.49 g of methacrylic acid were poured into the flask, then stirred for 10 min, then a 1 N NaOH solution was added to pH = 11 (pH meter pH-150MI, Izmeritelnaya Tekhnika LLC, Moscow, Russia). Then a pre-prepared mixture of comonomers was added to the flask: 0.49 g of methacrylic acid, 0.1 g of SSNa, and 3.3 g of styrene. The system was stirred for 15 min and then heated to 90 °C. Afterwards, 0.3 g of K_2_S_2_O_8_ as an initiator was added to the reaction system. The polymerization reaction was carried out for 1 h. Then the reaction system was cooled to room temperature. The synthesis of submicron particles SP-1 and SP-2 was also carried out by emulsifier-free emulsion copolymerization. The synthesis was carried out in a 100 mL flask equipped with a reflux condenser and a mechanical stirrer. 50 mL of a mixture of H_2_O:EtOH (40:60% vol.) was poured into the flask, and a pre-prepared mixture of comonomers was added: 0.15 g SSNa and 3.0 g styrene. The system was stirred for 15 min and then heated to 70 °C. Then 0.3 g of initiator (AIBN in the case of SN-1, K_2_S_2_O_8_ in the case of SN-2) was added to the reaction system. The polymerization reaction was carried out for 1 h. Then the reaction system was cooled to room temperature. The comparison particles MP-1 and MP-2 were synthesized by dispersion copolymerization. The synthesis was carried out in a 100 mL flask equipped with a reflux condenser and mechanical stirrer. 70 mL of EtOH was poured into the flask, 2 g of polymer stabilizer (polyvinylpyrrolidone-PVP) was added, and the mixture was stirred until the PVP was completely dissolved. Next, a pre-prepared comonomer mixture was added: 0.15 g of SSNa (or SPM), 1 mL of DMSO, and 26.5 g of styrene. The system was stirred for 15 min and then heated to 75 °C. Then, 0.6 g of CVA initiator was added to the reaction system. The polymerization reaction was carried out for 1 h. Then, the reaction system was cooled to room temperature. After polymerization of all comparison particles, polymerization by-products and unreacted monomers were removed by steam distillation and three consecutive centrifugation-redispersion cycles of the particles in bidistilled water (Centrifuge 5804, Eppendorf, Germany).

### 4.4. Particle Characterization

Freeze-dried samples were prepared and chemical composition analysis was performed using attenuated total reflection Fourier transform infrared (ATR-FTIR) spectroscopy (IRAffinity-1S spectrometer, Shimadzu, Kyoto, Japan). At least 30 spectra in the range of 600–2000 cm^−1^ were collected and averaged.

The shape and size distribution of swollen particles obtained was assessed using optical microscopy (MIKMED-5, Saint Petersburg, Russia). For better optical visualization, the polymer particles were contrasted with a solution of methylene blue. The optical images of microspheres were processed using the ImageJ (https://imagej.net/ij/download.html, accessed on 15 December 2025). The assessment of shape as well as surface layer morphology was also performed for freeze-dried particles using scanning electron microscopy (SEM) (SUPRA 55 VP microscope, ZEISS AG, Oberkochen, Germany). The hydrodynamic diameter of the resulting submicron particles and their polydispersity were determined by dynamic light scattering (DLS) (Zetasizer Nano-ZS analyzer, Malvern, UK).

The volume swelling coefficient (%) of the particles was determined as the ratio between the volume of the particles swollen in normal saline at 25 °C for 24 h until equilibrium and to the volume of the dry particle powder according to the formula published in [37].

The surface area and pore size distribution were evaluated from adsorption and desorption isotherms of N_2_ at −196 °C using an adsorption apparatus (NOVA Series 1200e analyzer, Quanta chrome, Boynton Beach, FL, USA) on freeze-dried samples by the Brunauer–Emmett–Teller method.

The ζ-potential of the particles in a 10^−3^ M NaCl aqueous solution was determined using a Malvern Nano ZS analyzer (Malvern, UK). To study the electrosurface properties of gel microparticles and comparison nano- and microparticles depending on the composition of the dispersion medium and pH, standard titers (ECROSKHIM Co., Ltd., Saint-Petersburg, Russia) were used; measurements of the ζ-potential of the particles were carried out after equilibration for 2 h.

The content of ionogenic groups was determined by conductometric titration and by measuring the ion-exchange capacity. To measure the ion-exchange capacity, a given weight of particles was dispersed in a estimated volume of 0.5 M HCl aqueous solution and kept for 24 h, then the particles were purified from excess acid by triple sequential cycles of “centrifugation−redispersion” (Centrifuge 5804, Eppendorf, Germany) in bidistilled water. Then the particles were lyophilized and redispersed in a 20 wt.% aqueous solution of NaCl and kept for 24 h. The acid released because of ion exchange was titrated under a flow of argon using 0.01 N NaOH aqueous solution (pH meter Mettler Toledo SevenMulti, Greifensee, Switzerland). Samples for conductometric titration were prepared in a similar manner by keeping the sample in a 1 mM aqueous NaCl solution. Data were recorded using a SevenMulti conductometer (MetlerToledo, Greifensee, Switzerland).

The gel fraction of porous particles was determined by continuous extraction in deionized water at 100 °C for 5 h using a Soxhlet apparatus (C.Gerhardt GmbH&Co.KG, Königswinter, Germany). Particles remaining after extraction was dried and weighted. Gel fraction was calculated as below:

$$Gel\;fraction\;\%=\frac{particle\;weight\;after\;extraction}{particle\;weight\;before\;extraction}\times 100\%$$

For the cytotoxicity assessment, human fetal mesenchymal stromal cells (FetMSCs) obtained from the Cell Culture Collection of the Institute of Cytology (St. Petersburg, Russia) were employed. The cell cultivation was performed in a humidified CO_2_ incubator at 37 °C with 5% CO_2_ in DMEM/F12 medium (Dulbecco’s Modified Eagle’s Medium; Biolot, Saint Petersburg, Russia) supplemented with 10% (*v*/*v*) heat-inactivated fetal bovine serum (FBS; HyClone, Logan, UT, USA), 1% L-glutamine, 50 U/mL penicillin, and 50 μg/mL streptomycin.

The cells were seeded at a density of 5.0 × 10^3^ cells per well in 100 μL of complete medium in 96-well plates. After 24 h, the culture medium was discarded and replaced with 100 μL of conditioned medium (particle extract) previously incubated with the experimental samples (particles were separated form extracts by centrifugation to exclude the influence of potential dye adsorption by gel microparticles on the results). The cells were then incubated for 72 h under standard culture conditions. Following this incubation period, the medium was removed, and 50 μL of fresh DMEM/F12 containing MTT (0.5 mg/mL) was added to each well. Cells were incubated for an additional 2 h at 37 °C in the CO_2_ incubator. The supernatant was then carefully removed, the intracellular formazan crystals formed by metabolically active cells were solubilized by adding 50 μL of dimethyl sulfoxide (DMSO) per well. The resulting solutions were transferred to new wells, and absorbance was measured at 570 nm using a microplate spectrophotometer AIFR-01 UNIPLAN (JSC PICON, Moscow, Russia). Data analysis was performed using polynomial regression in Microsoft Excel.

The % cytotoxicity was calculated as follows [38]:

% cytotoxicity = [(A − B)/A] × 100,

where A is the absorbance of the control and B is absorbance of the samples.

The % of cell viability was calculated as follows:

% viability = 100 − % cytotoxicity.

To study the adsorption of doxorubicin or fuchsine, weighted amount of microspheres was put into a bottle and aqueous solutions of absorbate with different concentration were added. The concentration of the absorbate in the supernatant was determined after 2 h by optical absorption at a wavelength of 480 nm (doxorubicin) or 550 nm (fuchsine), using a previously determined calibration curve.

## Figures and Tables

**Table 1:** Microspheres based on P(SSNa) and P(SPM) and their characteristic properties.

			Samples									
			No Comonomers		VA Comonomer		EA Comonomer		Crosslinker Mixture		+ Undecyl Alcohol	
			PSS-1	PSPM-1	PSS-2	PSPM-2	PSS-3	PSPM-3	PSS-4	PSPM-4	PSS-5	PSPM-5
Composition of the monomer mixture			SSNa 100	SPM 100	SSNa + VA 50:50	SPM + VA 50:50	SSNa + EA 50:50	SPM + EA 50:50	SSNa 100	SPM 100	SSNa 100	SPM 100
Property												
Yield, %			86	89	60	67	64	63	69	63	93	97
Gel fraction,%			97	91	89	88	96	85	92	95	92	88
D (80 wt. %), μm			0–19	0–8	0–12	0–16	0–6	0–15	0–8	0–23	0–4	0–17
Concentration of functional groups	ion exchange titration	mmol/g particles	2.4	2.2	2.1	1.6	1.7	1.8	2.7	3.4	3.0	2.5
		mmol/m^2^ particles	0.5	1.3	2.1	1.4	1.2	1.4	0.4	1.5	0.9	6.9
	conductometric titration	mmol/g particles	2.3	2.4	2.3	2.1	1.8	1.9	2.8	3.3	2.9	2.4
		mmol/m^2^ particles	0.5	1.4	2.3	1.8	1.2	1.5	0.4	1.5	0.8	6.6
Swelling degree, %			470	300	840	325	570	370	845	540	345	425
ξ-potential, mV (10^−3^ M NaCl)			−26	−34	−33	−28	−34	−29	−31	−46	−29	−28
Specific surface area, m^2^/g			4.61	1.71	0.99	1.17	1.48	1.29	7.17	2.23	3.54	0.363
Pore Volume, m^3^/g			0.005	0.002	0.001	0.002	0.002	0.002	0.008	0.003	0.004	0.001
Mesopore diameter, nm			3–12	3–11	3–11	2.9–13	3–11	3–15	2.7–11	2.7–12	3–12	2.9–14
Micropore presence			−	+	−	−	−	−	−	−	−	−

**Table 2:** Nano- and microparticles of comparison and their properties.

Property	Samples				
	NP1 P(St-SSNa)	SP1 P(St-SSNa)	SP2 P(St-SSNa)	MP1 P(St-SSNa)	MP2 P(St-SPM)
D, μm (SEM)	0.070	0.520	0.310	2–6	1–19
D, μm (DLS)	0.075	0.640	0.360	2–7	–
PDI	0.05	0.12	0.04	0.4	–
Concentration of functional groups (conductometric titration), mmol/g particles	0.24	0.18	0.19	0.039	0.027
ξ-potential, mV (10^−3^ M NaCl)	−65	−50	−47	−5	−7

**Table 3:** Maximum values of sorption capacity for gel microspheres and comparison particles in relation to doxorubicin.

	PSS1	PSS2	PSS3	PSPM1	PSPM2	PSPM3	SP1	SP2	MP1	MP2
	Nonlinear Langmuir model									
q_eq_ max. exp., mmol/g	2.22	1.86	1.65	1.86	1.97	1.85	0.45	0.20	0.0063	0.055
q_eq_ max. theor. Langmuir model, mmol/g	2.22	1.82	1.65	1.84	1.95	1.85	0.44	–	0.0085	0.108
R^2^	0.77	0.80	0.84	0.77	0.59	0.70	0.51	–	0.99	0.99
	Linear Freundlich model									
1/n	0.88	0.78	0.68	0.87	1.17	0.98	1.81	2.08	0.44	0.98
K_F_, mmol/g	1.29	1.48	1.50	1.71	2.54	2.09	20.56	56.88	58.33	1.71
R^2^	0.966	0.919	0.880	0.903	0.960	0.949	0.935	0.959	0.967	0.368

**Table 4:** Equivalent sorption capacity of gel microspheres based on P(SSNa) and P(SPM) with respect to fuchsine.

**q_eq_ Max., mmol/g**	**PSS-based Particles**				
	PSS-1	PSS-2	PSS-3	PSS-4	PSS-5
	3.6	2.9	2.5	3.3	3.5
	**SPM-based Particles**				
	SPM-1	SPM-2	SPM-3	SPM-4	SPM-5
	3.2	2.6	2.4	0.2	2.0

## Data Availability

The original contributions presented in this study are included in the article/Supplementary Material. Further inquiries can be directed to the corresponding authors.

## Supplementary Materials

The following supporting information can be downloaded at: https://www.mdpi.com/article/10.3390/ijms27010538/s1.
