# Supplementary material for: Gel Microparticles Based on Polymeric Sulfonates: Synthesis and Prospects for Biomedical Applications
Source: Int J Mol Sci. 2026 Jan 5;27(1):538. doi: 10.3390/ijms27010538 (PMC12786519; doi:10.3390/ijms27010538)
Supplement: Supplementary file 1 [file ijms-27-00538-s001.zip › ijms-4073254-supplementary.pdf]

Article

Gel microparticles based on polymeric sulfonates: synthesis and prospects for biomedical applications

Olga D. Iakobson[1], Elena M. Ivan'kova[1], Yulia Nashchekina[2] and Natalia N. Shevchenko[1]*

[1] Branch of Petersburg Nuclear Physics Institute named by B.P. Konstantinov of National Research Centre
«Kurchatov Institute»—Institute of Macromolecular Compounds, 199004 Saint-Petersburg, Russia 1;
[2] Institute of Cytology, Russian Academy of Sciences, 194064 Saint-Petersburg, Russia
* Correspondence: iakobson_od@pnpi.nrcki.ru (O.I.); shevchenko_nn@pnpi.nrcki.ru (N.S.)

Supporting information

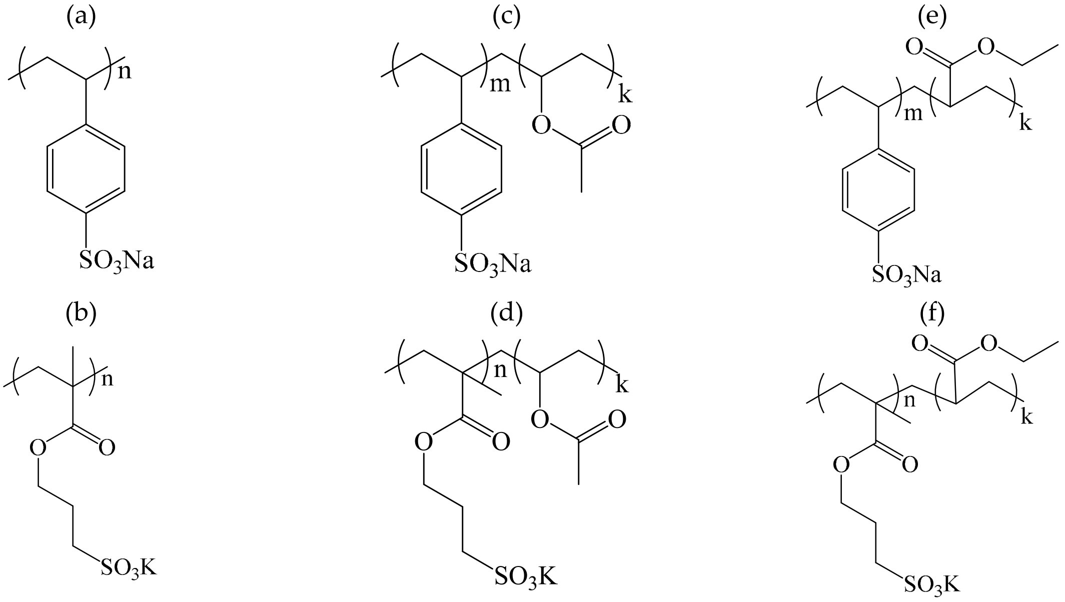

Figure S1. Chemical formulas of sodium polystyrene sulfonate (a, P(SSNa)), potassium polysulfopropyl methacrylate (b, P(SPM)) and their copolymers with vinyl acetate (c, P(SSNa-co-VA); d, P(SPM-co-VA)) and ethyl acrylate (e, P(SSNa-co-EA); f, P(SPM-co-EA)).

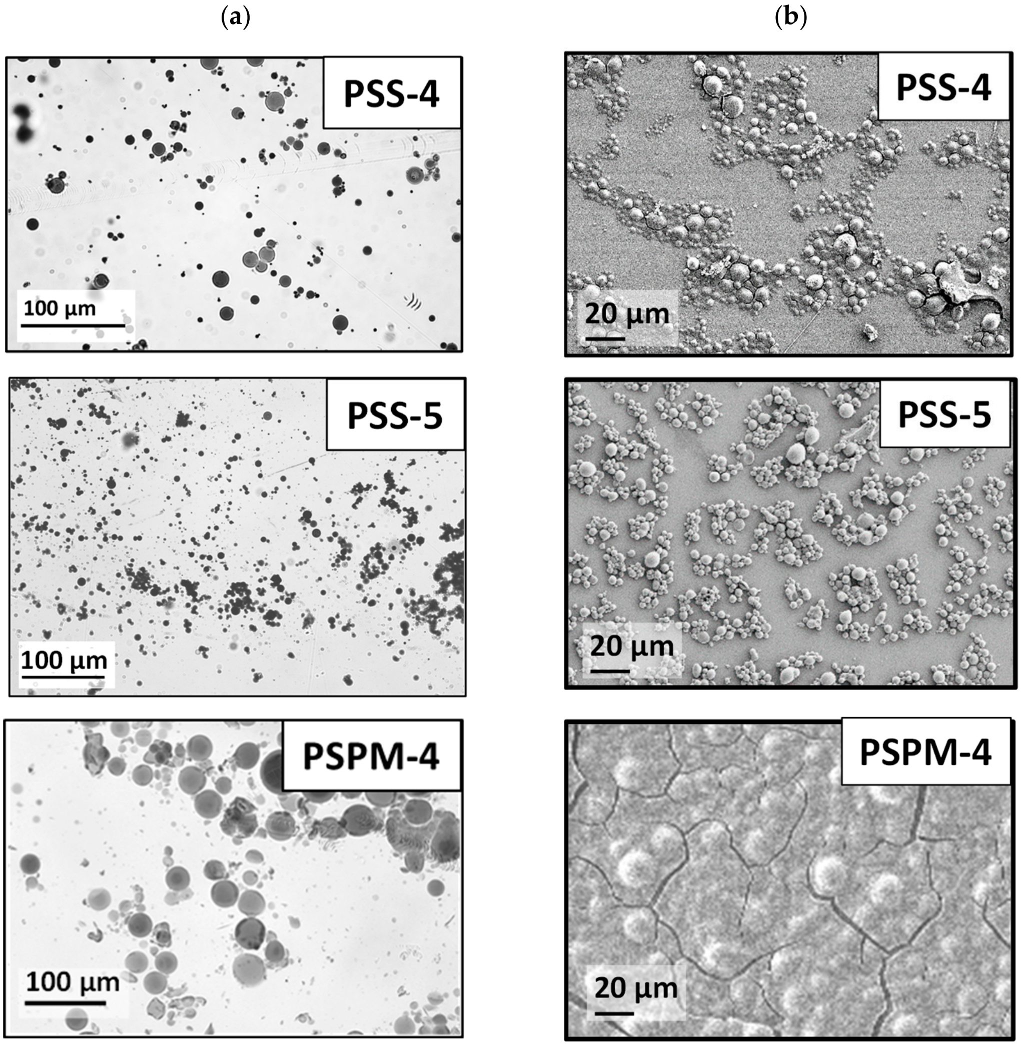

Figure S2. Images of microspheres based on P(SSNa) and P(SPM) obtained by optical microscopy (a) and scanning electron microscopy (b).

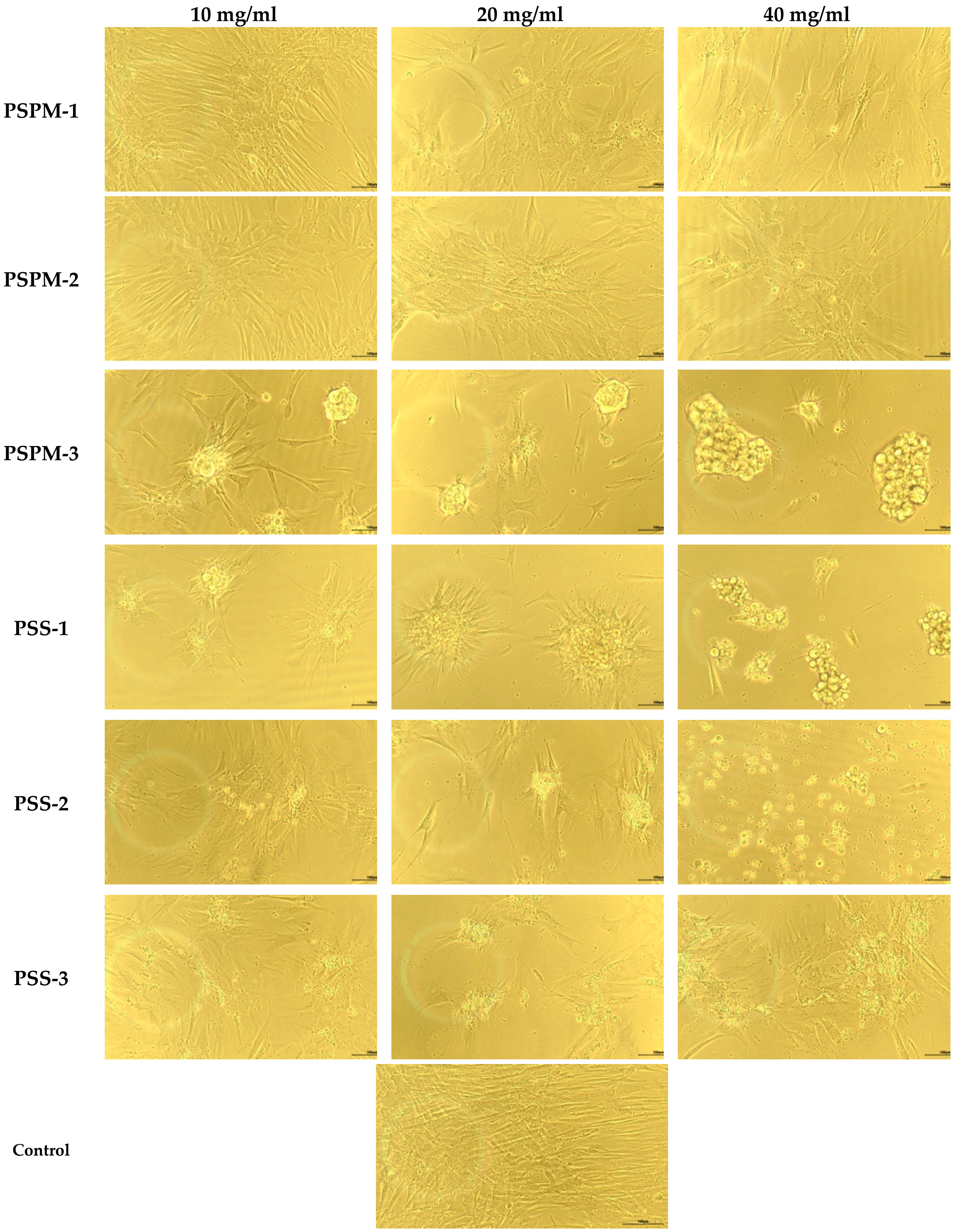

Figure S3. Optical microscopy images of cells in the presence of medium after 3 days of cultivation after incubation with gels microparticles.

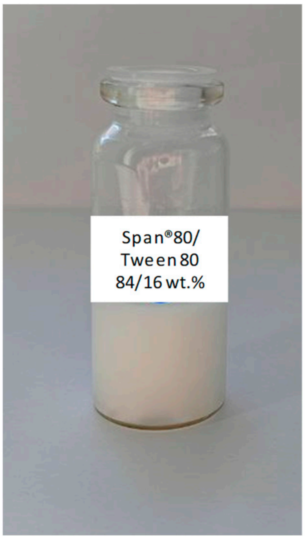

Figure S4. Visual appearance of the water-in-oil emulsion stabilized by Span® 80/Tween 80 (86:14, w/w) after 24 h of emulsification.
